# Cases Admitted under the Care of the Surgeon of the Finsbury Dispensary, St. John's Square, Clerkenwell, from November 10, to December 10, 1802

**Published:** 1803-01-01

**Authors:** J. Ricards

**Affiliations:** No. 68, Hatton Street


*Cases admitted under the Care of the Surgeon of the Finsbury Dispensary, St. John's Square, Clerkenwell, from November* 10, *to December* 10, 1802.

| | | | |
|---|---|---|---|
| Phlegmone | 2 | Vulnera Artuum | 3 |
| Erysipelas | 6 | Contusiones | 2 |
| Arthrodynia | 1 | Combustura | 6 |
| Abscessus Artuum | 2 | Lues | 4 |
| Mastodynia | 1 | Gonorrhœa | 1 |
| Abscessus Scrophulosi | 7 | Strictura Urethra | 1 |
| Ulcera Artuum | 12 | Crusta Lactea | 2 |
| ——— Faucium | 2 | Eruptiones Chronicæ | 4 |
| Fistulæ in Perinæo | 2 | | |
| Cancer Uteri | 1 | Total | 58 |

There has been less *variety* in the diseases of our Report during the last Month than usual, as will appear from the above list, which is principally, with a very few exceptions, composed of inflammatory diseases and their consequences, and of accidents: indeed, our attention has been very much confined to a single disease under a variety of forms, and arising from various causes; which has been so extremely frequent in our district of late, as justly to acquire the denomination of an epidemic; I allude to Erysipelas. Although, in the above list, there appears only six cases under that name, which includes merely those which arose idiopathically, without any manifest external cause, yet almost every case of wound, ulcer, &c. &c. has been succeeded by, or attended with, inflammation of this description, to a more or less extent.

I have observed, in the progress of this disease, that some of the most dangerous cases have arisen from the most (apparently) trivial causes: Several instances of wounded fingers and whitloes have been followed by Erysipelatous inflammation to such extent, as to occasion considerable alarm with respect to the life of the patient. One case, which was attended with a peculiar rapidity of progress, and malignity of appearance, was in the person of a woman, about fifty years of age, of a habit, feeble, delicate, and extremely susceptible of irritation: She had a whitloe form in the thumb, which was small; this was opened by a lancet, when the formation of matter was evident. A few days after, the glands at the elbow and and axilla became enlarged, and an inflammatory blush appeared over those parts, and along the course of the absorbents; this was very soon followed by an universal Erysipelas of the whole limb, from the fingers to the shoulder, and even extending along a part of the trunk. In the course of a very few hours from the appearance of the Erysipelas, (so rapid was its progress) vesications arose on every part, several of which were as large as hens eggs, and of a livid aspect: The system was affected with *extreme debility* and symptoms of irritation. By the very copious administration of *cinchona, opium,* and cordials, with spirituous local applications, the disease was subdued, and the patient is now nearly recovered.

This is not the only case wherein causes as slight as the above have produced erysipelatous affections of great danger; they have arisen frequently around old ulcers, from wounds of (primarily) no apparent consequence; and in several instances, have appeared without any known cause. One case of the latter kind occurred in the leg of a man, who was, though young, of an originally delicate habit; which state of constitution had been increased by his occupation, which was that of a painter. After a rigor, Erysipelas attacked his whole lower extremity; and very shortly proceeded to a state of gangrene, and nearly the whole of the integuments of the leg and foot sloughed; he, nevertheless, recovered.

With particular attention I have remarked, that every case of Erysipelas which has occurred, has been, unequivocally, a disease of debility. The majority of the objects of charity in a large city, are of debilitated constitutions, and peculiarly subject to debile diseases from their habits of life; but this observation is not universal, as there are some of vigorous constitutions and robust stamina: The former of these are liable to the attacks of Erysipelas; the latter to phlegmonous inflammation; from the same remote causes manifesting an analogy with the different species of fever, Synocha and Typhus. This assertion will appear accurate, by comparing the above case of Erysipelas succeeding a disease of the finger, (of which kind several other cases have occurred) which happened in habits of debility, with two other recent cases which occurred in contrary constitutions. Two young men, of robust and healthy appearance, had each a whitloe of the finger; in both, inflammation extended along the absorbents, (as in the above case of Erysipelas) and the ulnar and axillary glands became enlarged; but in this state of the disease, the inflammation in these did not spread, spread, like Erysipelas, over the whole arm, but confined itself to a particular spot. In one of these cases the glands, just above the elbow, inflamed; in the other, one of the axillary glands: that inflammation was truly phlegmonous, and in both cases suppurated, forming buboes, which readily healed.

There was a great similarity in cause and effort of Nature in all these cases: The cause was alike in all; the absorbent vessels in all were inflamed, and the glands were slightly enlarged. In the healthy and vigorous constitutions, the healthy inflammation took place in the glands; an inflammation which works its own cure: but in the debilitated habits, Nature was insufficient to effect this operation; and the inflammation of disease and debility took place instead; an inflammation which the system is scarcely able to sustain, requiring the most vigorous aids which we are enabled to bestow.

It were scarcely necessary to mention a fact, which, I believe, is by most practitioners admitted, that Erysipelas is a disease of debility, and requires treatment of the most tonic and invigorating nature, but that some modern writers have directed the contrary mode of practice. It appears to me that it is probable, that there must be a difference in the nature of the disease, in different local situations, when we read* that "It is now known, that blood may be discharged with the same safety in Erysipelas as in other cases of inflammation." And that we should adhere "in every respect to an antiphlogistic regimen." And that, by so doing, "almost every Erysipelatous tumor may be discussed." I would by no means think of contradicting these assertions, as in all probability they are strictly facts, as far as regards the country in which this author practises; but in this city I have seldom, if ever, seen a case of Erysipelas, in which, from the habit of the patient, or other contingent symptoms, I should have dared to have made use of the lancet: on the contrary, I have never observed more decided and rapid advantage result from the administration of medicine, in any disease, than I have seen from the use of the cinchona, wine, opium, and other cordial tonics, in very considerable doses, in every variety of Erysipelas.

*No. 68, Hatton Street.*

J. RICARDS.

---

* Bell's System of Surgery, 6th Edition, Vol. I. p. 128.

*Account*

